# *catena*-Poly[[diaqua­bis(thio­cyanato-κ*N*)cobalt(II)]-μ-4,4′-bipyridine-κ^2^ *N*:*N*′] 4,4′-bipyridine solvate]

**DOI:** 10.1107/S1600536809023265

**Published:** 2009-06-24

**Authors:** Rufu Yao, Dong E. Wang

**Affiliations:** a Department of Chemistry, Hefei Teachers College, Hefei, Anhui 230061, People’s Republic of China; b The Department of chemistry, Kashgar Teachers College, Kashgar, Xinjiang 844000, People’s Republic of China

## Abstract

In the title complex, {[Co(NCS)_2_(C_10_H_8_N_2_)(H_2_O)_2_]·C_10_H_8_N_2_}_*n*_, the Co^II^ ion is located on an inversion centre and is coordinated by two N atoms from the two 4,4′-bipyridine ligands, two O atoms from the water mol­ecule, and two N atoms from two isothio­cyanate ions in a distorted octa­hedral environment. In the crystal, the coordinated water mol­ecules, isothio­cyanate ions and solvent 4,4′-bipyridine mol­ecules are linked by O—H⋯S and O—H⋯N hydrogen bonds into layers parallel to the *ab* plane.

## Related literature

For two-dimensional Mn^II^ and one-dimensional Cu^II^ complexes constructed from 4,4′-bipy, see: Yang *et al.* (2008 ▶); Zhou & He (2008 ▶). For related structures, see: Lu *et al.* (1997 ▶); He *et al.* (2006 ▶). 
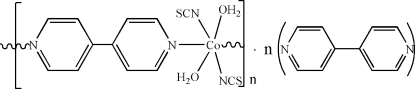


## Experimental

### 

#### Crystal data

[Co(NCS)_2_(C_10_H_8_N_2_)(H_2_O)_2_]·C_10_H_8_N_2_
*M* *_r_* = 523.51
Triclinic, 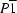

*a* = 7.4433 (11) Å
*b* = 9.0147 (11) Å
*c* = 10.1114 (13) Å
α = 107.770 (2)°
β = 103.978 (2)°
γ = 97.038 (2)°
*V* = 612.66 (14) Å^3^
*Z* = 1
Mo *K*α radiation
μ = 0.90 mm^−1^
*T* = 293 K
0.20 × 0.20 × 0.20 mm

#### Data collection

Bruker SMART CCD area-detector diffractometer
Absorption correction: multi-scan (*SADABS*; (Bruker, 2001 ▶) *T*_min_ = 0.835, *T*_max_ = 0.835
3522 measured reflections
2359 independent reflections
2191 reflections with *I* > 2σ(*I*)
*R*_int_ = 0.012

#### Refinement

*R*[*F* ^2^ > 2σ(*F* ^2^)] = 0.042
*wR*(*F* ^2^) = 0.118
*S* = 1.04
2359 reflections
157 parameters
3 restraints
H atoms treated by a mixture of independent and constrained refinement
Δρ_max_ = 0.77 e Å^−3^
Δρ_min_ = −0.74 e Å^−3^

### 

Data collection: *SMART* (Bruker, 2001 ▶); cell refinement: *SAINT* (Bruker, 2001 ▶); data reduction: *SAINT*; program(s) used to solve structure: *SHELXS97* (Sheldrick, 2008 ▶); program(s) used to refine structure: *SHELXL97* (Sheldrick, 2008 ▶); molecular graphics: *SHELXTL* (Sheldrick, 2008 ▶); software used to prepare material for publication: *SHELXTL*.

## Supplementary Material

Crystal structure: contains datablocks I, global. DOI: 10.1107/S1600536809023265/kp2215sup1.cif

Structure factors: contains datablocks I. DOI: 10.1107/S1600536809023265/kp2215Isup2.hkl

Additional supplementary materials:  crystallographic information; 3D view; checkCIF report

## Figures and Tables

**Table 1:** Selected bond lengths (Å)

Co1—N2	2.089 (2)
Co1—O1	2.0964 (19)
Co1—N5	2.1625 (18)

Symmetry code: (i) 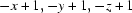.

**Table 2:** Hydrogen-bond geometry (Å, °)

*D*—H⋯*A*	*D*—H	H⋯*A*	*D*⋯*A*	*D*—H⋯*A*
O1—H1*C*⋯N7	0.82 (3)	1.92 (3)	2.732 (3)	171 (3)
O1—H1*B*⋯S1^ii^	0.82 (3)	2.52 (3)	3.279 (2)	154 (3)

Symmetry code: (ii) 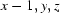.

**Fig. 1.**
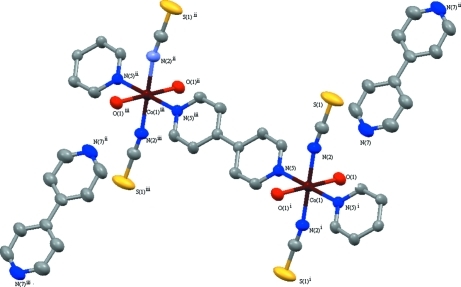
A part of polymeric chain of CoII octahedra with 4,4-bpy bridging ligand is shown with ellipsoids at the 30% probability level. Symmetry codes used: (i) -x + 1,-y + 1,-z + 1; (ii) x - 1, y, z. (iii)-x, -y, -z.

**Fig. 2.**
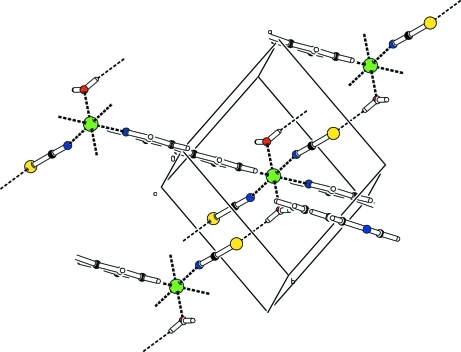
The crystal packing of (I) with hydrogen bonds (dashed lines).

## supplementary crystallographic information

### Comment

For 2-D Mn^II^ and 1-D Cu^II^ complexes constructed from 4,4'-bipy see: Yang *et al.* (2008) and Zhou, *et al.* (2008). The compound of [Co(4,4'-bipy)(NCS)_2_(OH_2_)_2_].(4,4'-bipy) were reported (Lu, *et al.* 1997) but the polymeric compound has not been synthesized, so far. Herein, we report the crystal structure of a novel polymeric compound, {[Co(4,4'-bipy)(NCS)_2_(OH_2_)_2_].(4,4'-bipy)}_n_. The Co^II^ ion is coordinated by two N atoms from 4,4'-bipy ligand, two N atoms from isothiocyanate ions, and two O atoms from two water molecule in a distorted octahedral geometry (Fig. 1, Table 1). O(1), N(2), O(1)^i ^and N(2)^i^[symmetry code: -1 + *x*, *y*, *z*] lie in the equatorial plane, with the O(1)—N(2)—O(1)^i^—N(2)^i^ torsional angle of 0.02 (11)°, while the Co atom deviates from the equatorial plane by 0.051 Å. N(5), N(5)^i^ atoms occupy the axial sites, which are strictly linear due to a symmetry opeartion. The bond lengths and angles of the title complex are similar to the compound {[Co(4,4'-bipy)(ambdc)(OH_2_)_2_](4,4'-bipy)(DMF)}_n_ (He *et al.*,2006). In the crystal packing of (*l*) are linked by O—H···S and O—H···N hydrogen bonds (Table 2, Fig. 2).

### Experimental

A mixture of Co(CH_3_COO)_2_(0.5 mmol), 4,4'-bipy (0.5 mmol and H_2_O (10.00 ml), was placed in a Parr Teflon-lined stainless steel vessel (10 ml), and then the vessel was sealed and heated at 393 K for 3 d. After the mixture was slowly cooled to room temperature, a few red crystals of [Co(4,4'-bipy)(NCS)_2_(OH_2_)_2_].(4,4'-bipy) were obtained.

### Refinement

H atoms of water molecule were located in a difference map and refined with O—H distance restraints of 0.82 (3)) Å, and *U*_iso_(H) = 1.5*U*_eq_(O). Other H atoms bonded to C atoms were introduced at calculated positions and refined using a riding model, with *U*_iso_(H) = 1.2*U*_eq_(C), and C—H distances of 0.93 Å.

### Crystal data

**Table:**

[Co(NCS)_2_(C_10_H_8_N_2_)(H_2_O)_2_]·C_10_H_8_N_2_	*V* = 612.66 (14) Å^3^
*M**_r_* = 523.51	*Z* = 1
Triclinic, *P*1	*F*(000) = 269
Hall symbol: -P 1	*D*_x_ = 1.419 Mg m^−^^3^
*a* = 7.4433 (11) Å	Mo *K*α radiation, λ = 0.71073 Å
*b* = 9.0147 (11) Å	θ = 1.0–26.0°
*c* = 10.1114 (13) Å	µ = 0.90 mm^−^^1^
α = 107.770 (2)°	*T* = 293 K
β = 103.978 (2)°	Block, red
γ = 97.038 (2)°	0.20 × 0.20 × 0.20 mm

### Data collection

**Table:**

Bruker SMART CCD area-detector diffractometer	2359 independent reflections
Radiation source: fine-focus sealed tube	2191 reflections with *I* > 2σ(*I*)
graphite	*R*_int_ = 0.012
φ and ωs scans	θ_max_ = 26.0°, θ_min_ = 2.2°
Absorption correction: multi-scan (*SADABS*; Sheldrick, 1997)	*h* = −9→4
*T*_min_ = 0.835, *T*_max_ = 0.835	*k* = −11→11
3522 measured reflections	*l* = −12→12

### Refinement

**Table:**

Refinement on *F*^2^	Primary atom site location: structure-invariant direct methods
Least-squares matrix: full	Secondary atom site location: difference Fourier map
*R*[*F*^2^ > 2σ(*F*^2^)] = 0.042	Hydrogen site location: inferred from neighbouring sites
*wR*(*F*^2^) = 0.118	H atoms treated by a mixture of independent and constrained refinement
*S* = 1.04	*w* = 1/[σ^2^(*F*_o_^2^) + (0.0744*P*)^2^ + 0.3257*P*] where *P* = (*F*_o_^2^ + 2*F*_c_^2^)/3
2359 reflections	(Δ/σ)_max_ < 0.001
157 parameters	Δρ_max_ = 0.77 e Å^−^^3^
3 restraints	Δρ_min_ = −0.74 e Å^−^^3^

### Special details

**Table:**

Geometry. All e.s.d.'s (except the e.s.d. in the dihedral angle between two l.s. planes) are estimated using the full covariance matrix. The cell e.s.d.'s are taken into account individually in the estimation of e.s.d.'s in distances, angles and torsion angles; correlations between e.s.d.'s in cell parameters are only used when they are defined by crystal symmetry. An approximate (isotropic) treatment of cell e.s.d.'s is used for estimating e.s.d.'s involving l.s. planes.
Refinement. Refinement of *F*^2^ against ALL reflections. The weighted *R*-factor *wR* and goodness of fit *S* are based on *F*^2^, conventional *R*-factors *R* are based on *F*, with *F* set to zero for negative *F*^2^. The threshold expression of *F*^2^ > σ(*F*^2^) is used only for calculating *R*-factors(gt) *etc*. and is not relevant to the choice of reflections for refinement. *R*-factors based on *F*^2^ are statistically about twice as large as those based on *F*, and *R*- factors based on ALL data will be even larger.

### Fractional atomic coordinates and isotropic or equivalent isotropic displacement parameters (Å^2^)

**Table:**

	*x*	*y*	*z*	*U*_iso_*/*U*_eq_	
Co1	0.5000	0.5000	0.5000	0.03359 (17)	
S1	0.93240 (12)	0.61882 (16)	0.23801 (12)	0.0837 (4)	
O1	0.3666 (3)	0.6731 (2)	0.4436 (2)	0.0453 (4)	
H1B	0.251 (3)	0.659 (4)	0.418 (4)	0.068*	
H1C	0.403 (4)	0.719 (4)	0.394 (3)	0.068*	
N2	0.6824 (3)	0.5256 (3)	0.3768 (2)	0.0467 (5)	
N5	0.2992 (3)	0.3143 (2)	0.3142 (2)	0.0365 (4)	
C1	0.2050 (4)	0.1832 (3)	0.3238 (3)	0.0439 (6)	
H1A	0.2201	0.1757	0.4154	0.053*	
C2	0.0868 (4)	0.0590 (3)	0.2048 (3)	0.0433 (6)	
H2A	0.0247	−0.0295	0.2172	0.052*	
C3	0.0605 (3)	0.0664 (3)	0.0661 (2)	0.0326 (5)	
C4	0.1567 (4)	0.2036 (3)	0.0576 (2)	0.0390 (5)	
H4	0.1423	0.2157	−0.0322	0.047*	
C5	0.2730 (4)	0.3217 (3)	0.1814 (2)	0.0398 (5)	
H5	0.3369	0.4115	0.1721	0.048*	
C21	0.7868 (4)	0.5654 (3)	0.3206 (3)	0.0439 (6)	
N7	0.4547 (4)	0.8112 (3)	0.2551 (3)	0.0652 (7)	
C11	0.5596 (5)	0.9552 (4)	0.2955 (4)	0.0664 (8)	
H11	0.6222	1.0072	0.3939	0.080*	
C12	0.5829 (5)	1.0347 (4)	0.2006 (3)	0.0579 (7)	
H12	0.6588	1.1365	0.2356	0.070*	
C13	0.4917 (4)	0.9604 (3)	0.0537 (3)	0.0458 (6)	
C14	0.3805 (5)	0.8079 (4)	0.0096 (4)	0.0590 (7)	
H14	0.3170	0.7522	−0.0881	0.071*	
C15	0.3664 (6)	0.7408 (4)	0.1137 (4)	0.0695 (9)	
H15	0.2901	0.6397	0.0828	0.083*	

### Atomic displacement parameters (Å^2^)

**Table:**

	*U*^11^	*U*^22^	*U*^33^	*U*^12^	*U*^13^	*U*^23^
Co1	0.0348 (3)	0.0325 (3)	0.0224 (2)	−0.00978 (17)	0.00294 (17)	0.00493 (17)
S1	0.0513 (5)	0.1408 (10)	0.0912 (7)	0.0135 (5)	0.0309 (5)	0.0798 (7)
O1	0.0453 (10)	0.0441 (10)	0.0408 (10)	−0.0031 (8)	0.0058 (8)	0.0172 (8)
N2	0.0450 (12)	0.0515 (12)	0.0351 (11)	−0.0079 (10)	0.0114 (10)	0.0105 (9)
N5	0.0366 (10)	0.0335 (10)	0.0271 (9)	−0.0071 (8)	0.0014 (8)	0.0052 (8)
C1	0.0494 (14)	0.0412 (13)	0.0265 (11)	−0.0144 (11)	0.0032 (10)	0.0067 (10)
C2	0.0469 (14)	0.0385 (12)	0.0303 (11)	−0.0154 (10)	0.0048 (10)	0.0067 (10)
C3	0.0293 (10)	0.0321 (11)	0.0271 (10)	−0.0005 (9)	0.0022 (9)	0.0048 (9)
C4	0.0471 (13)	0.0326 (11)	0.0266 (10)	−0.0036 (10)	−0.0002 (10)	0.0087 (9)
C5	0.0451 (13)	0.0311 (11)	0.0318 (11)	−0.0072 (10)	0.0011 (10)	0.0087 (9)
C21	0.0379 (13)	0.0521 (15)	0.0370 (12)	−0.0010 (11)	0.0043 (10)	0.0180 (11)
N7	0.0780 (18)	0.0692 (17)	0.0740 (18)	0.0243 (15)	0.0358 (15)	0.0466 (15)
C11	0.078 (2)	0.072 (2)	0.0585 (19)	0.0154 (18)	0.0229 (17)	0.0335 (17)
C12	0.0635 (18)	0.0549 (17)	0.0609 (18)	0.0066 (14)	0.0204 (15)	0.0285 (14)
C13	0.0490 (14)	0.0465 (14)	0.0560 (15)	0.0158 (11)	0.0268 (12)	0.0266 (13)
C14	0.073 (2)	0.0491 (16)	0.0597 (18)	0.0059 (14)	0.0237 (16)	0.0255 (14)
C15	0.085 (2)	0.0537 (18)	0.082 (2)	0.0077 (17)	0.033 (2)	0.0361 (17)

### Geometric parameters (Å, °)

**Table:**

Co1—N2^i^	2.089 (2)	C3—C4	1.388 (3)
Co1—N2	2.089 (2)	C3—C3^ii^	1.486 (4)
Co1—O1	2.0964 (19)	C4—C5	1.374 (3)
Co1—O1^i^	2.0964 (19)	C4—H4	0.9300
Co1—N5	2.1625 (18)	C5—H5	0.9300
Co1—N5^i^	2.1625 (18)	N7—C11	1.318 (5)
S1—C21	1.630 (3)	N7—C15	1.330 (5)
O1—H1B	0.817 (18)	C11—C12	1.390 (4)
O1—H1C	0.813 (17)	C11—H11	0.9300
N2—C21	1.151 (3)	C12—C13	1.382 (4)
N5—C5	1.333 (3)	C12—H12	0.9300
N5—C1	1.341 (3)	C13—C14	1.394 (4)
C1—C2	1.378 (3)	C13—C13^iii^	1.490 (5)
C1—H1A	0.9300	C14—C15	1.382 (4)
C2—C3	1.393 (3)	C14—H14	0.9300
C2—H2A	0.9300	C15—H15	0.9300
			
N2^i^—Co1—N2	180.0	C3—C2—H2A	120.1
N2^i^—Co1—O1	90.16 (9)	C4—C3—C2	116.3 (2)
N2—Co1—O1	89.84 (9)	C4—C3—C3^ii^	121.7 (2)
N2^i^—Co1—O1^i^	89.84 (9)	C2—C3—C3^ii^	122.0 (3)
N2—Co1—O1^i^	90.16 (9)	C5—C4—C3	120.3 (2)
O1—Co1—O1^i^	180.0	C5—C4—H4	119.9
N2^i^—Co1—N5	88.84 (8)	C3—C4—H4	119.9
N2—Co1—N5	91.16 (8)	N5—C5—C4	123.5 (2)
O1—Co1—N5	90.36 (7)	N5—C5—H5	118.3
O1^i^—Co1—N5	89.64 (7)	C4—C5—H5	118.3
N2^i^—Co1—N5^i^	91.16 (8)	N2—C21—S1	178.8 (3)
N2—Co1—N5^i^	88.84 (8)	C11—N7—C15	116.2 (3)
O1—Co1—N5^i^	89.64 (7)	N7—C11—C12	124.3 (3)
O1^i^—Co1—N5^i^	90.36 (7)	N7—C11—H11	117.8
N5—Co1—N5^i^	180.0	C12—C11—H11	117.8
Co1—O1—H1B	121 (2)	C13—C12—C11	119.1 (3)
Co1—O1—H1C	121 (2)	C13—C12—H12	120.4
H1B—O1—H1C	106 (3)	C11—C12—H12	120.4
C21—N2—Co1	169.0 (2)	C12—C13—C14	117.1 (3)
C5—N5—C1	116.7 (2)	C12—C13—C13^iii^	121.9 (3)
C5—N5—Co1	120.28 (15)	C14—C13—C13^iii^	121.0 (3)
C1—N5—Co1	122.85 (15)	C15—C14—C13	118.8 (3)
N5—C1—C2	123.3 (2)	C15—C14—H14	120.6
N5—C1—H1A	118.3	C13—C14—H14	120.6
C2—C1—H1A	118.3	N7—C15—C14	124.4 (3)
C1—C2—C3	119.9 (2)	N7—C15—H15	117.8
C1—C2—H2A	120.1	C14—C15—H15	117.8

Symmetry codes: (i) −*x*+1, −*y*+1, −*z*+1; (ii) −*x*, −*y*, −*z*; (iii) −*x*+1, −*y*+2, −*z*.

### Hydrogen-bond geometry (Å, °)

**Table:**

*D*—H···*A*	*D*—H	H···*A*	*D*···*A*	*D*—H···*A*
O1—H1C···N7	0.82 (3)	1.92 (3)	2.732 (3)	171 (3)
O1—H1B···S1^iv^	0.82 (3)	2.52 (3)	3.279 (2)	154 (3)

Symmetry codes: (iv) *x*−1, *y*, *z*.
